# Symptom severity of depressive symptoms impacts on social cognition performance in current but not remitted major depressive disorder

**DOI:** 10.3389/fpsyg.2015.01118

**Published:** 2015-08-04

**Authors:** Tracy Air, Michael J. Weightman, Bernhard T. Baune

**Affiliations:** Discipline of Psychiatry, School of Medicine, The University of AdelaideAdelaide, SA, Australia

**Keywords:** major depressive disorder, social cognition, facial affect, prosody, depressive symptoms

## Abstract

The aim of the present study was to investigate the social cognitive functioning of participants with depression when compared with healthy controls, and to assess the impact of symptom severity. One hundred and eight patients with depression (66 remitted and 42 current) and 52 healthy controls were assessed using the Wechsler Advanced Clinical Solutions: Social Perception Subtest, measuring facial affect recognition in isolation and in combination with prosody and body language interpretation. When healthy controls, remitted depression and currently depressed groups were compared, no associations were found on any of the social cognition subscales. Severity of depressive and anxious symptoms predicted performance on all social cognition subscales in currently depressed participants, controlling for age, gender, education and psychotropic medication. Affective depressive symptoms were inversely related to ACS Pairs and Prosody subscales, while somatic symptoms were inversely related to the ACS Affect Recognition and Total scores. There was no association between severity and the WAIS ACS in remitted depression participants. People with MDD exhibiting more severe depressive and anxious symptoms and a cluster of affective symptoms have greater difficulty undertaking complex social cognitive tasks. Given the state like nature to these deficits, these impairments may cause problems with day to day functioning and have implications in targeted therapeutic interventions.

## Introduction

Individuals with major depressive disorder (MDD) have reported pervasive impairments in cognitive functioning, including difficulties in attention (Douglas and Porter, 2009; Rock et al., 2013), memory loss (McDermott and Ebmeier, 2009), reduced psychomotor functioning (Sobin and Sackeim, 1997; Lecrubier, 2006) and social cognition (Tse and Bond, 2004).

Social cognition can be defined as the mental operations that underlie social interactions, including perceiving, interpreting, and generating responses to the intentions, dispositions, and behaviors of others (Green et al., 2008). This can include emotion-processing, theory of mind, and social perception and knowledge.

The relationship between social cognition and depression is somewhat ambiguous (Weightman et al., 2014). Numerous studies have found that depressed patients demonstrate significantly poorer social cognition compared with healthy controls (Surguladze et al., 2004; Langenecker et al., 2005; Csukly et al., 2009; Szily and Kéri, 2009; Harkness et al., 2011; Van Wingen et al., 2011). For example, there have been consistent positive findings in the theory of mind literature, particularly in tasks requiring participants to correctly interpret an affective mental state portrayed in images of eyes. The clear majority of studies found that depressed patients were impaired in this skill compared to controls (Lee et al., 2005; Wang et al., 2008; Szily and Kéri, 2009; Harkness et al., 2011; Cao et al., 2013). Additionally, in research investigating emotion perception, depressed individuals are significantly more likely to interpret a neutral stimulus as being sad, and assign more negative interpretations to neutral expressions than healthy controls (Leppänen et al., 2004; Milders et al., 2010; Anderson et al., 2011). Also, depressed patients have been found to need greater intensity of emotion than their non-depressed counterparts when asked to identify happy expressions, and less intensity to identify sad expressions (Gollan et al., 2008, 2010; Milders et al., 2010). However, other studies have found no group difference between depressed patients and controls on a variety of measures of social cognition, including prosodic stimuli (Kan et al., 2004), visual perception (Bazin et al., 2009), and affect recognition (Joormann and Gotlib, 2006; Suslow et al., 2010; Bertoux et al., 2012).

A reason for such disparity in the field could be because the relationship between social cognition and other cognitive process has not been widely or comprehensively studied (Pessoa, 2009; Millan et al., 2012). Studies into disorders such as schizophrenia, ADHD and bipolar disorder have shown that the processes behind social cognition can be partially explained by processing speed (Anselmetti et al., 2009; Dhar et al., 2010; Antila et al., 2011) and executive function (Kerns et al., 2008; Torralva et al., 2011). Given that executive function and information processing have been shown to been detrimentally effected in MDD patients (Fossati et al., 2002; Tsourtos et al., 2002; Baune et al., 2010; Snyder, 2013), it is a reasonable to assume that these would also have an impact social cognition also. While emotional information processing research has been gaining traction in the past decade (Suslow et al., 2004; Gilboa-Schechtman et al., 2005; Karparova et al., 2005), the relationship between executive function and social cognition requires further research (Uekermann et al., 2008; Ladegaard et al., 2014; Thoma et al., 2015).

It has also been suggested that the disparity in these findings may stem from the use of a wide variety of social cognition measures (Weightman et al., 2014), different social cognitive paradigms, depression severity or differing clinical presentations (Cusi et al., 2012). While consensus on paradigms or social cognitive measures may be insurmountable, numerous studies have investigated the role of symptom severity. For example, Johnson and Dilorenzo (1998) found that dysphoric female students were less accurate than female non-dysphoric students when identifying positive interpersonal reactions when viewing videotapes of dating scenarios but tended to be more accurate when identifying negative reactions. Donges et al. (2005) demonstrated that depressed patients showed a reduced emotional awareness for other persons. Emotional awareness for others increased as the depression symptoms abated, however they still did not reach the levels of healthy controls. Finally, as depressive symptoms increased, recognition accuracy increased for sad faces, but decreased for other emotions such as surprise (Gollan et al., 2010).

In addition to severity of symptoms, a small body of research has also investigated the impact of the clinical presentation of symptoms on social cognition. Lee et al. (2005) reported that affective depression symptoms, such as anhedonia, had a significant inverse relationship with performance in a theory of mind task. Suicidal behavior has also been found to strongly correlate with the impaired interpretation of social stimuli (Wang et al., 2008; Szanto et al., 2012; Cao et al., 2013), as has excessive rumination (Raes et al., 2006).

Most of the findings described above were obtained in current MDD patients, whereas little is known about social cognitive biases following recovery from a depressive episode. The scar hypothesis proposes that people develop maladaptive characteristics that linger even after a depressive episode has ended, putting them at greater risk for future depressive episodes (Lewinsohn et al., 1981). Those with MDD may develop a certain dysfunctional interpersonal style, as they are at greater risk for being socially rejected than non-depressed people (Hammen, 1991; Marcus and Nardone, 1992; Segrin and Abramson, 1994). Even after recovery, they may continue to exhibit particular beliefs or behaviors in social situations (Abela et al., 2009). Some authors have specifically examined whether currently depressed patients performed differently on social cognitive tasks compared to those with remitted major depressive disorder and healthy controls. Evidence supporting the scar hypothesis in remitted MDD patients has been found particularly in the area of facial emotion recognition. Remitted MDD patients are more likely to identify anger and fear when compared to controls (Bhagwagar et al., 2004; Anderson et al., 2011), while Lemoult et al. (2009) found that patients with remitted MDD required significantly greater emotional intensity to identify happy expressions than controls.

The aims of this study were three-fold: first, to assess performance of participants with a history of depression vs. healthy controls on social cognition measures, to determine whether or not there were deficits in social cognition in the MDD group. Second, to investigate the validity of the scar hypothesis with respect to social cognition; that is, to explore potential differences between healthy controls, remitted MDD and current MDD on measures of facial affect recognition, prosody and elements of theory of mind. The final aim was to assess the extent to which the severity of depressive symptomatology contributes to the effects of MDD on social cognition, in the depressed sample only. In line with the literature that emotion processing is impaired in depression, we also explored the concept that affective symptoms will heighten the association between depression severity and social cognitive performance. In particular, we investigated the relationships between affective and somatic subsets of depression symptoms with social cognitive performance.

## Materials and methods

### Study design

A case-control design was employed to recruit participants with a mood disorder and healthy controls, with the mood disorder group further divided into currently depressed and remitted depressed groups. Current depression was defined as those participants currently depressed as per the MINI 6.0.0, while remitted depression was defined as a participants with a prior (although not current) history of depression based on the MINI 6.0.0.

Approval for the project was gained from the human research ethics committees of the University of Adelaide and Royal Adelaide Hospital. Inclusion criteria for the study were (1) aged 15 or over, and (2) adequate reading and writing skills and the ability to speak the English language. Exclusion criteria included diagnosis of schizophrenia, dementia, a learning disorder, eating disorder, or a Pervasive Developmental Disorder (e.g., autistic spectrum disorder). In total, 160 subjects met the inclusion criteria and were included for analysis.

### Participants

MDD participants were recruited from the general community and clinical services within the Eastern Mental Health network, in Adelaide, South Australia. Advertisements were placed throughout the Royal Adelaide Hospital, The University of Adelaide campus, and local community noticeboards to allow further recruitment.

Healthy controls were recruited via word of mouth from the general community, or again through posters advertisements placed throughout the Royal Adelaide Hospital, The University of Adelaide campus, and local community noticeboards. The sample was comprised of 52 healthy controls, 66 participants with remitted MDD, and 42 participants with current MDD.

### Measures

#### Psychiatric interview

All participants were assessed with the MINI-6.0.0 Neuropsychiatric Diagnostic Interview (Sheehan et al., 1998). The MINI is a short diagnostic tool that takes approximately 30–45 min to administer. It is designed to generate 17 Diagnostic and Statistical Manual DSM-IV or ICD 10 Axis I diagnoses and has good to excellent specificity and sensitivity concordance to both the Structured Clinical Interview for the American Psychiatric Association Diagnostic criteria (SCID), and the Composite International Diagnostic Interview (ICD-10) (Sheehan et al., 1998). The MINI has been widely used in more than 100 studies and translated into more than 30 different languages.

#### Depressive symptom severity measure

Participants were also administered the Structured Interview Guide for the Hamilton Depression and Anxiety Scales (SIGH-AD; Williams, 1988) to assess symptom severity. The SIGH-AD is a 31-item questionnaire that combines a 17-item depression scale (HAM-D) with a 14-item anxiety scale (HAM-A). Each item relates to a particular group of symptoms in the week prior to interview.

#### Social cognitive assessment

Social cognition was assessed using the novel *Wechsler Advanced Clinical Solutions: Social Perception Subtest* (WAIS-ACS) (Pearson, 2009). This is an integrated test including facial affect, prosody, body language and mental state interpretation. It has the advantage of a large normative sample of 800 subjects matched to the US census (Holdnack et al., 2011; Kandalaft et al., 2012). Subtest raw scores of the ACS use a scaled score metric, with a mean of 10 and a standard deviation of 3. The WAIS-ACS comprises of:

- Affect recognition (ACS Affect): This section involved showing the participant multiple pictures of male and female faces expressing one of seven different emotions—happiness, sadness, anger, fear, disgust, surprise, or neutral—and instructing them to select the correct emotion expressed from the above list. The maximum raw score for this subscale is 24.
- Prosody-face matching (ACS Prosody): This subset involved playing the participant a recorded voice and instructing them to match the emotional tone of the speaker with one of six faces displaying various expressions (the seven emotions from Affect Naming with sarcasm and confusion added). The maximum raw score for this subscale is 24.
- Prosody-pair matching (ACS Pairs): For this task, the participant again listened to a recorded statement and selected the most appropriate picture for the tone of the speaker from four different pictures of two actors interacting. After selecting a picture, the participant was asked to describe the emotion expressed by the speaker, and to determine whether the tone of voice used altered the meaning of the comment (such as with sarcasm). If so, they were required to state the real meaning behind the speaker's comment. The maximum raw score for this subscale is 42.
- Social Perception Total Score (ACS Total): The Social Perception Total score is derived by adding all of the correct matches of pictures of one to two people from the affect naming and prosody tasks, with a raw maximum score of 48.

### Statistical analysis

Statistical analyses were performed using Stata, version 12 (Stata Statistical Software: Release 12. College Station, TX: StataCorp LP). Categorical variables were analyzed using chi-squared tests and continuous variables were analyzed using One-Way ANOVA. Adjustments for multiple comparisons were not performed, as these corrections have been criticized for masking potentially important findings in clinical research (Perneger, 1998). Due to the exploratory nature of this study, the authors decided that the risk of making a type I error was less important than the risk of Type II error.

ANOVAs were conducted to identify differences between the healthy controls, the remitted depression and current depression groups for the Affect Naming subscale, Prosody subscale, Pairs subscale and Social Perception Total Score. Age, gender, years of education and use of psychotropic medication were controlled for. Multivariate linear regression models were utilized to assess the association between the social cognition measures (Affect Naming, Prosody, Pairs and Social Perception Total Score) and symptom severity as assessed by the SIGH-AD. Age, gender, years of education and use of psychotropic medication were controlled for in the linear regression analyses. In the first step of the multiple regression analysis, the covariates were entered into the model. In the second step, the covariates plus the main effects of depression, anxiety or total symptom severity were entered into the model.

Two depressive symptom clusters were also composed from the SIGH-AD (Steer et al., 1987; Lee et al., 2005) by summing scores of individual items to generate continuous scores. The first scale, affective depression, was comprised of summed scores of depressed mood, guilt, suicide, work and activities, and retardation. The second scale, somatic depression, comprised of early, middle, and late insomnia, anxiety-somatic, somatic-gastrointestinal, somatic-general, genital symptoms, hypochondriasis, and weight loss. The continuous variables representing the two clusters were then both entered into a multivariate linear regression analysis to assess their relationship with the social cognition scales. Again, age, gender, years of education and use of psychotropic medication were controlled for.

## Results

### Demographics

Table 1 displays demographic and clinical data for the sample. The study groups did not differ in gender or years of education. The healthy controls were significantly younger (*t* = −2.66, *df* = 157, *p* = 0.009), and significantly less likely have a family history of MDD (χ = 18.8, *df* = 1, *p* < 0.001) or have a current prescription for psychotropic medication (χ = 34.4, *df* = 1, *p* < 0.001). Significant differences in symptom severity were found between MDD and healthy control participants for the SIGH-AD Depression (*t* = −9.1, *df* = 156, *p* < 0.001), SIGH-AD Anxiety (*t* = −9.0, *df* = 156, *p* < 0.001) and SIGH-AD total score (*t* = −9.4, *df* = 156, *p* < 0.001). One-Way ANOVAs revealed there was a significant difference between healthy controls, remitted depression and currently depressed groups on the SIGH-AD depression [*F*_(2, 154)_ = 101.01, *p* < 0.001] and anxiety [*F*_(2, 154)_ = 79.55, *p* < 0.001] subscales. As expected, *post-hoc* Scheffe's tests showed that patients with current depression had significantly higher SIGH-AD depression scores compared with the healthy controls (*p* < 0.001) and the remitted depression group (*p* < 0.001), significantly higher SIGH-AD anxiety scores (Healthy controls: *p* < 0.001; Remitted: *p* < 0.001), and significantly higher SIGH-AD total scores (Healthy controls: *p* < 0.001; Remitted: *p* < 0.001).

**Table 1:** **Demographic and clinical characteristics**.

	**Healthy Controls**	**Remitted MDD**	**Acute MDD**	**HC vs. Rem MDD**	**HC vs. Acute MDD**	**Overall**
	**(*n* = 52)**	**(*n* = 66)**	**(*n* = 42)**	***p*-values^b^**	***p*-values^b^**	***p*-values^a^**
Age (mean ± SD)	28.3 ± 16.7	35.4 ± 17.1	37.4 ± 17.4	0.07	0.04	0.02
Gender (n, %)						
Females	26 (50.0)	39 (59.1)	30 (71.4)	NA	NA	0.10
Years of education (mean ± SD)	13.1±1.9	13.3±1.9	12.9±1.9	0.67	0.79	0.44
Family history of major depressive disorder (*n*, %)						
Yes	13 (25.0)	41 (62.1)	26 (61.9)	NA	NA	< 0.001
Currently using psychotropic medication (*n*, %)						
Yes	0 (0.0)	23 (34.9)	26 (61.9)	NA	NA	< 0.001
SIGH-AD (mean ± SD)						
Depression	3.5 ± 2.9	9.8 ± 5.7	18.3 ± 6.5	< 0.001	< 0.001	< 0.001^b^
Anxiety	2.8 ± 2.6	9.6 ± 5.9	17.4 ± 7.5	< 0.001	< 0.001	< 0.001^b^
Total	6.3 ± 5.2	19.3 ± 11.1	35.8 ± 13.1	< 0.001	< 0.001	< 0.001^b^

a *P-values obtained from One-Way ANOVAs for continuous variables and Chi-Square test for binary variables*.

b *Scheffe's post-hoc test*.

### Current vs. remitted MDD group differences in social cognition

There were no differences between the healthy controls, the remitted group or the currently depressed group on any of the ACS subscales or the total score (Table 2). The means and standard deviations for all groups are within the normal range, and inspection of the frequency distributions did not demonstrate any evidence of a ceiling effect. The overall depressed sample did not differ significantly on any of the social cognition measures when compared with the healthy controls. Further analyses, comparing healthy controls with remitted MDD and current MDD, also revealed no significant differences on the social cognition measures.

**Table 2:** **Means and standard deviations of the standardized ACS SP subscales, among participants with Major Depressive Disorder (MDD) and healthy controls**.

	**Healthy controls (*n* = 52)**	**Lifetime MDD (*n* = 108)**	***p*-values^a^**	**Remitted MDD (*n* = 66)**	**Acute MDD (*n* = 42)**	**HC vs. Rem MDD *p*-values^b^**	**HC vs. Acute MDD *p*-values^b^**	**Overall *p*-values^b^**
ACS affect recognition	10.1 ± 3.0	10.0 ± 2.8	0.91	10.0 ± 2.7	10.0 ± 3.0	0.87	0.45	0.89
ACS prosody face-matching	11.5 ± 2.7	10.8 ± 2.6	0.13	10.7 ± 2.5	10.8 ± 2.7	0.29	0.82	0.31
ACS prosody pair matching	11.3 ± 3.1	11.0 ± 2.9	0.54	11.1 ± 2.8	10.7 ± 3.1	0.64	0.99	0.61
ACS total score	11.2 ± 3.0	10.6 ± 2.9	0.28	10.5 ± 2.7	10.8 ± 3.0	0.32	0.47	0.51

a *P-values obtained from students t-test for independent groups*.

b *P-values obtained from ANOVAs with pairwise comparisons of adjusted means, adjusted for age, gender, years of education and use of psychotropic medication controlled for*.

*ACS Affect Recognition, participants shown photos of facial expressions and asked to identify one of seven different emotions; ACS Prosody Face Matching, Participants listen to a recorded voice and asked to match the emotional tone of the speaker with one of six faces; ACS Prosody Pair Matching, Participants listen to a recorded voice and select an appropriate picture and emotion from four different pictures of two actors interacting; ACS Total Score, Derived by adding all of the correct matches of pictures from the affect naming and prosody tasks*.

### Relationship between symptom severity and social cognition

**Figure 1:**
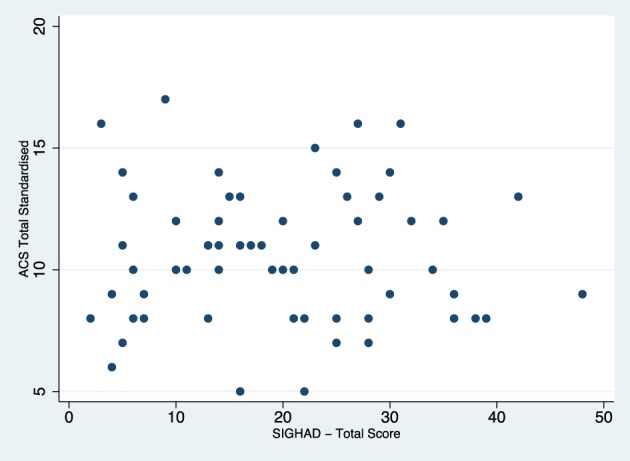
**Scatterplot of SIGH-AD and total ACS scores for the remitted depression group**.

**Figure 2:**
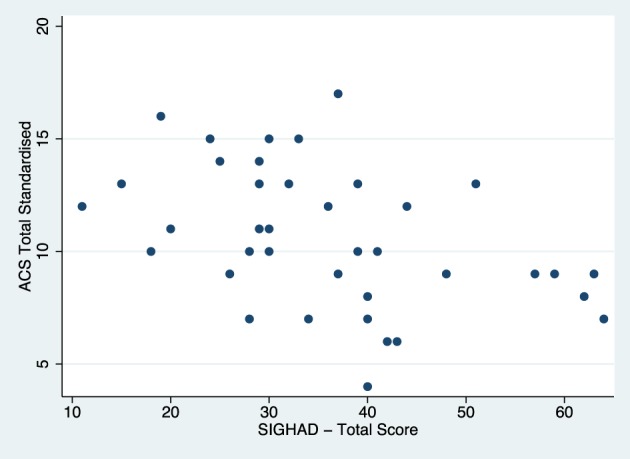
**Scatterplot of SIGH-AD and total ACS scores for the acute depression group**.

Both the ACS and SIGH-AD total scores were assessed for restriction of variance separately in the current and remitted groups. Figures 1, 2 display the scatterplots for the ACS total score and the SIGH-AD. Neither scatterplot shows evidence of restriction of variance.

Multivariate linear regression analyses were performed in the overall depressed sample to assess the impact of symptom severity on social cognition (Table 3). In the overall depressed sample, performance on the ACS Prosody was significantly inversely associated with SIGH-AD Anxiety and Total scores; and the ACS Pairs test was significantly inversely associated with SIGH-AD Anxiety, SIGH-AD Depression and Total scores. The association between the ACS total score, SIGH-AD Anxiety and SIGH-AD Total approached significance.

**Table 3:** **Multivariate linear regression models for assessing the relationship between depression severity and social cognition performance in acute and remitted major depressive disorder (MDD)**.

	**ACS affect recognition**		**ACS prosody face-matching**		**ACS prosody pair-matching**		**ACS total score**	
	**β^a^**	**p^b^**	**β^a^**	**p^b^**	**β^a^**	**p^b^**	**β^a^**	**p^b^**
**ALL MDD CASES**								
SIGH-AD Anxiety	−0.06	0.12	−0.09	0.02	−0.11	0.01	−0.08	0.051
SIGH-AD Depression	−0.06	0.15	−0.08	0.06	−0.12	0.005	−0.07	0.11
SIGH-AD Total	−0.04	0.12	−0.05	0.02	−0.06	0.007	−0.04	0.05
								
**ACUTE MDD CASES ONLY**								
SIGH-AD Anxiety	−0.18	0.009	−0.15	0.04	−0.18	0.02	−0.17	0.01
SIGH-AD Depression	−0.21	0.007	−0.19	0.02	−0.27	0.003	−0.22	0.01
SIGH-AD Total	−0.13	0.003	−0.10	0.02	−0.12	0.008	−0.12	0.005
**REMITTED MDD CASES ONLY**								
SIGH-AD Anxiety	0.02	0.74	−0.11	0.07	−0.08	0.22	−0.05	0.42
SIGH-AD Depression	0.10	0.89	−0.06	0.33	−0.08	0.21	−0.03	0.63
SIGH-AD Total	0.10	0.81	−0.05	0.15	−0.04	0.20	−0.02	0.50

a *Denotes standardized beta*.

b *P-values obtained from linear regression analyses, adjusted for age, gender, years of education, psychotropic medication. ACS Affect Recognition, participants shown photos of facial expressions and asked to identify one of seven different emotions; ACS Prosody Face Matching, Participants listen to a recorded voice and asked to match the emotional tone of the speaker with one of six faces; ACS Prosody Pair Matching, Participants listen to a recorded voice and select an appropriate picture and emotion from four different pictures of two actors interacting; ACS Total Score, Derived by adding all of the correct matches of pictures from the affect naming and prosody tasks*.

Further diagnostic group stratified analyses showed that higher scores on the SIGH-AD scale (total scores and anxiety and depression subscales) in the currently depressed group were all significantly associated with poorer social cognitive scores on all ACS subscales and the ACS total score. In contrast, no significant effects of the SIGH-AD scores on social cognition were found in the remitted depression subgroup.

### Effects of affective and somatic symptom clusters on social cognition

Finally, analysis of the affective and somatic symptom clusters revealed significant relationships with the ACS subscales and total score in the currently depressed group. The affective symptom subscale had a significant inverse relationship with both the ACS Pairs (standardized β = −0.44, *SE* = 0.17, *p* = 0.014) and Prosody (standardized β = −0.34, *SE* = 0.15, *p* = 0.033), while the somatic subscale had a significant inverse relationship with ACS affect recognition (standardized β = -0.58, *SE* = 0.6, *p* = 0.001) and Total score (standardized β = −0.50, *SE* = 0.17, *p* = 0.007).

## Discussion

The present study investigated social cognitive functioning in a sample of healthy controls and participants with current or remitted MDD. Impairments on several social cognitive domains were not observed when depressed individuals regardless of the currency of symptoms were compared to control subjects. However, our results suggest that certain characteristics of a currently depressed and anxious state are responsible for the social performance deficits observed in some mood disorder populations, with severity being one of these factors.

However, multivariate regression analyses demonstrated significant associations between symptom severity and social cognition in the MDD group that were currently depressed. Additionally, significant relationships were found between depressive symptom clusters and the ACS subscales and total score in the group they were currently depressed.

The literature is divided over the influence of diagnostic categories in social cognition. While a number of studies have found that depressed patients demonstrate significantly poorer performance on social cognition measures when compared with healthy controls (Leppänen et al., 2004; Surguladze et al., 2004; Langenecker et al., 2005; Csukly et al., 2009), there have been other studies that found no differences (Bediou et al., 2005; Matthews et al., 2008; Gollan et al., 2010; Suslow et al., 2010). Depressed participants in our study did not differ from healthy controls in either recognition of facial or auditory affect. Our results suggest that if sufficient information is available, depressed participants did not perform significantly worse from controls in their interpretation of facial and prosody affect. However, level of depressive symptoms relate to poorer ACS scale performance only in current depression. As has been found in this study, both clusters or intensity of illness symptomatology may provide important clinical correlates that are more likely to be specifically associated with deficits in social functioning, irrespective of diagnosis.

Furthermore, the finding that there were no relationships between symptom severity and the ACS in the remitted group alone yet such relationships existed in the currently depressed group, suggest that patients with MDD display recovery in social cognition after symptomatic remission, contrary to the scar hypothesis. Previous research has found inconsistent evidence for a scarring effect—Lemoult et al. (2009) reported that patients with remitted major depressive disorder made fewer errors on facial affect recognition than controls, but needed significantly greater intensity to identify certain emotions. Other studies have found that patients in remission were more likely to identify fear (Bhagwagar et al., 2004) or anger (Anderson et al., 2011). It is possible that our study may not have assessed the social cognition variables most likely to show scarring effects, or perhaps the effect is more subtle. It could be that social cognition in remitted patients is mediated by other factors such as general day to day functioning or neurocognition in general, or is a mediator itself, as has been hypothesized in the psychosis literature (Ventura et al., 2009; Lin et al., 2013).

It has also been suggested that the phenotype of depression could be important, with affective symptoms identified as having a greater effect on performance than neurovegetative features (Lee et al., 2005). Our research adds to the body of research that has demonstrated a relationship between specific depression symptoms and deficits in social cognitive performance. Given the relationship between the affective symptom subscale and the more complicated prosody subscales of the ACS, this could provide support for the hypothesis put forth by Lee et al. (2005). In participants suffering from current depression, affective symptoms including anhedonia and hopelessness could mean that they are unlikely to allocate cognitive resources to attending to and processing social information. We also found that currently depressed participants who scored higher on the somatic symptoms performed worse on the affect recognition task. Recent studies have demonstrated that somatoform patients recognized a significantly lower proportion of emotional expressions than did the healthy controls (Pedrosa Gil et al., 2009; Pollatos et al., 2011). Pedrosa Gil and colleagues suggested that impaired facial emotion recognition might be a general feature of somatoform disorders, Pollatos et al. (2011) reported that while healthy controls reacted to an emotion recognition task with an increase in parasympathetic nervous system activity, this “normal” reactivity pattern was affected in somatoform disorder patients. It is possible that this may not be restricted to somatoform disorder patients, and that currently depressed patients who experience a high level of somatic symptoms could react in a similar way.

There were a number of limitations with the study. One of the main limitations of the study is that motivation, IQ, overall cognitive function or, at the very least, subdomains of cognitive function, were not assessed and therefore could not be controlled for. This is of particular importance given that, in depressive patients, brain dysfunctions have been reported in regions that are assumed to be critical for emotional processing and cognitive (particularly executive) functions (Cusi et al., 2012). However, a recent study by Ladegaard et al. (2014) found that controlling for basic cognitive function did not change significant differences between healthy controls and a MDD group on the social cognitive tasks. The authors concluded that social cognition may be an independent domain for which non-social cognition represents a necessary but not sufficient condition. Another limitation of this study was that the depression group was heterogeneous in composition, incorporating patients with a single historical major depressive episode as well as those with repeated episodes of depression. Also, while the *ACS-SP* is a useful and unique tool, it is an American test, making it difficult to assess utility into an Australian context, particularly for prosody. While the recorded voices were of American accents, there were no colloquialisms unfamiliar to Australians. Finally, the *ACS-SP* test did not have standardized scores for performance on particular valences of emotion. This is widely performed in the literature and would have indicated if certain emotions showed greater deficits, even if not reflected in the overall score. It is therefore difficult to comment on the negative interpretative bias from these results.

### Implications for future research

The key question arising from this investigation is whether impairments with social cognition causes problems with functioning or quality of life over and above that which is already associated with depression. Finally, further studies need to elucidate whether social cognition is impaired by both emotion-processing deficits as well as by cognitive deficits within the same study. These questions are beyond the scope of the present study, but are important in order to establish the clinical significance of the findings.

## Conclusions

The results suggest that in a currently depressed state, as symptom severity increases, people with MDD have more difficulties in processing complex social visual and auditory cues. It also appears that specific symptom clusters are likely to be associated with aspects of impaired social cognition. Further study is needed to determine the relationship between social cognition, overall cognition, depression and functioning.

## Author contributions

TA collected data, analyzed data and wrote a first draft. MW collected data, managed data and performed first analyses. BB conceived the study, the study design and revised and edited manuscript drafts. All authors approved the final manuscript.

### Conflict of interest statement

The authors declare that the research was conducted in the absence of any commercial or financial relationships that could be construed as a potential conflict of interest.
